# Patient and family experience with transthyretin amyloid cardiomyopathy (ATTR-CM) and polyneuropathy (ATTR-PN) amyloidosis: results of two focus groups

**DOI:** 10.1186/s13023-021-01706-7

**Published:** 2021-02-08

**Authors:** David Rintell, Dena Heath, Florencia Braga Mendendez, Elizabeth Cross, Theodore Cross, Vincent Knobel, Bruno Gagnon, Cameron Turtle, Alan Cohen, Edward Kalmykov, Jonathan Fox

**Affiliations:** 1 BridgeBio, Palo Alto, CA USA; 2 Amyloidosis Research Consortium, Newton, MA USA; 3 Alianza Argentina de Pacientes, Buenos Aires, Argentina; 4 Cross Associates, Woodland Park, NJ USA; 5 Eidos Therapeutics, San Francisco, CA USA

## Abstract

**Background:**

Transthyretin amyloidosis, or ATTR, is a progressive and debilitating rare proteopathy generally manifested as either transthyretin amyloid polyneuropathy (ATTR-PN) or transthyretin amyloid cardiomyopathy (ATTR-CM). Irrespective of the clinical presentation, affected patients manage a chronic and life-threatening condition that severely impacts their quality of life. Although the primary symptoms and diagnostic criteria for ATTR are increasingly being discussed in the medical literature, due in large part by continual advances in uncovering disease pathophysiology, there exists a surprising paucity of published data on the patient journey and family experience. In order to address this disparity, two focus groups, one for ATTR-CM and one for ATTR-PN, were convened and asked to describe the diagnostic process, symptoms, and impact on their own quality of life that was experienced from these rare and typically misdiagnosed illnesses.

**Results:**

Patients in both ATTR groups often underwent a long and difficult diagnostic odyssey characterized by seemingly nonspecific physical manifestations resulting in mismanagement and suboptimal care, inadequate interventions, and delays in establishing the correct diagnosis, which was integral to determining the specialized treatment they needed. Collectively, patients with ATTR-CM and patients with ATTR-PN reported a similar number of symptoms, but the type of symptoms varied. The ATTR-CM group identified intolerance to activity, inability to exercise, insomnia and fatigue as the most challenging symptoms. The ATTR-PN group identified fatigue, diarrhea/constipation and sensory deficits as the most difficult symptoms. In general, ATTR was reported to be highly stressful for both patients and their families. Spouses of patients with ATTR-CM were often in a caregiver role and reported experiencing considerable anxiety. Patients with ATTR-PN were stressed not only by the physical consequences of their illness, but also by its effects on their parents and other relatives, as well as concerns about children and grandchildren inheriting the disease-causing mutations associated with ATTR. Despite such challenges, family members are identified as an important resource of coping, motivation, inspiration and support.

**Conclusions:**

Several steps can be taken to reduce the challenges and burdens of living with ATTR, including increased education for primary care physicians and specialists who unknowingly encounter ATTR, increased access to and ready availability of mental health services and support, and increased engagement with support groups and advocacy organizations. Input from patients and their representatives should guide clinical trials, increase the availability of genetic testing, and generate natural history and qualitative studies detailing patients’ experience. Although each recommendation is impactful in itself, taken together they would jointly facilitate a shortened and ameliorated patient journey through more timely diagnosis and greater access to personalized medical care.

## Background

Systemic amyloidosis is a family of rare diseases that manifest due to protein misfolding and deposition into a variety of organ systems, thus leading to a range of chronic and progressive disorders. There are more than 15 types of systemic amyloidosis, each the byproduct of different precursor proteins promoting amyloid formation and tissue deposition. Amyloidosis can be acquired or hereditary and can impact a variety of vital organs, including the heart, nerves, gastrointestinal tract, kidneys, lungs, liver, muscles and skin [1].

Symptoms attributable to amyloidosis are usually nonspecific and insidious, often resulting in delayed or missed diagnosis. Despite significant improvements in non-invasive diagnostic modalities and accuracy within the past decade, even when combined with the emergence of new classes of therapy and management strategies, the patient journey for those suffering with amyloidosis remains arduous and frustrating due in large part to the collective rarity and nonspecific clinical presentations seen across the condition’s various types [2].

One member of this family of syndromes is transthyretin amyloidosis, also known as ATTR or ATTR amyloidosis, which is caused by the dissociation of the transthyretin protein tetramer into its constituent monomers and subsequent accumulation as misfolded and aggregated protein deposits, or amyloid, in and around organs and tissues [3, 4]. Although amyloid deposition is indiscriminate and therefore may affect any part of the body, the most prevalent areas leading to distinct pathologies are around the peripheral nerves, resulting in transthyretin amyloid polyneuropathy, or ATTR-PN, and the heart, leading to a related yet different form known as transthyretin amyloid cardiomyopathy, or ATTR-CM [1].

Transthyretin amyloid polyneuropathy (ATTR-PN) is a predominantly genetic form of the illness characterized by a generalized, length-dependent (or ascending) peripheral neuropathy involving the sensory, motor, and autonomic nervous systems. The variable genetic disposition of ATTR-PN results in either early manifestations, with an average onset being 30 years of age, or occurring later in life around or after 50 years. Although the true global prevalence continues to remain unclear, estimates place the incidence of ATTR-PN between 10,000 to 40,000 persons at the upper limit [5, 6].

Transthyretin amyloid cardiomyopathy (ATTR-CM) is the result of amyloid deposits in the myocardium causing progressive heart failure. It can occur in individuals with or without inherited mutations [7, 8]. As with ATTR-PN, the heritable form of ATTR-CM (ATTRm-CM) is characterized by both endemic distribution and differential onset ranging from 30 to 80 years of age depending on the mutation. The non-heritable, or wild-type, form (ATTRwt-CM) typically presents after the 6th decade of life with the average foundational age being approximately 75 years and is much more pervasive and globally ubiquitous [9]. Long thought to be an uncommon disorder, emerging findings purport that ATTR-CM, particularly the wild-type variety, will become the dominant diagnosed form of cardiomyopathy resulting from amyloidosis with a variable yet ever-increasing estimated global prevalence [11, 13]. Despite the etiological differences in ATTR presentation, all forms of transthyretin amyloidoses (wild type—ATTRwt-CM and hereditary/mutant ATTRm-CM or ATTR-PN) lead to reduced functionality (effort tolerance and activities of daily living) and premature death [8].

The symptoms and diagnostic criteria of ATTR amyloidosis are increasingly well established and agreed upon in the medical literature, which is primarily written by and for sub-specialists in academic settings and specialty clinics. Nevertheless, despite current changes in diagnostic criteria and recommended “best practices,” many community cardiologists and primary care physicians remain unaware of relevant clinical and scientific advances due to the rarity of the disease and the nonspecific symptomatology [8, 14–17].

In addition to the measurably distinct clinical parameters commensurate with assessing the severity of a given disease, there also exist subjective multidimensional aspects that reflect the uniquely individual and personal journey each patient undergoes as the disease shapes and alters their life and circumstances, generally referred to as quality of life, or QOL [10]. Investigations into the QOL of people living with ATTR typically employ approaches utilizing validated quantitative instruments (e.g., Kansas City Cardiomyopathy Questionnaire [14, 19]; Norfolk Quality of Life Questionnaire-Diabetic Neuropathy [15, 20, 21]; EuroQOL 5-Dimention 3-Level Instrument [18]), as utilized by several recent interventional clinical trials examining disease-modifying agents as well as observational studies and investigations aimed at uncovering ATTR natural history [12]. Such inquiries have reported lower QOL for people living with ATTR as compared to the general population and to people living with other long-term, chronic diseases including multiple sclerosis, diabetic neuropathy, and irritable bowel syndrome [16, 22]. Not surprisingly, QOL scores in ATTR have also been reported to decrease with worsening illness [23, 24]. Despite escalating awareness and agreement on ATTR-CM and PN diagnostic criteria within the sub-specialist healthcare community, the timely diagnoses of ATTR often remains delayed due to misdiagnosis or incorrect attribution of the multiplicity of symptoms to more common medical conditions.

Alongside quantifiable measures and evaluations ascertaining the effects of ATTR on the quality of patients’ lives, there also exist nuanced qualitative aspects that are equally informative, particularly with respect to the overall disease experience. Nonetheless, outside of this investigation, we know of no previous qualitative studies on the nature of patients’ lived experience with the illness. The Amyloidosis Research Consortium’s report on testimony to the FDA does, however, include a highly useful thematic analysis of patient reports [26]. Qualitative research can inform treatment providers about patients’ needs and identify opportunities for improvements in patient care. This qualitative study catalogs and reports the responses from two focus groups comprised of patients with ATTR-CM and ATTR-PN and their family caregivers provides new insights into the real-world experiences of those living directly and indirectly with ATTR.

## Methods

In collaboration with two patient organizations: the Northern California Amyloidosis Support Group (affiliated with the Amyloidosis Research Consortium), and the Alianza Argentina de Pacientes, we convened a focus group for ATTR-CM patients and family members in San Francisco, California, and ATTR-PN patients and their family members in Buenos Aires, Argentina. The ATTR-CM group included the following: 4 male patients with wild-type ATTR cardiomyopathy (ATTRwt-CM), 2 male patients and 1 female patient with hereditary (mutant) ATTR cardiomyopathy (ATTRm-CM), and the partners of three of the patients in the group. The ATTR-PN focus group included 10 patients and 5 family members. Several of the ATTR-PN patients also had relatives (including their children) with ATTR-PN who they referenced in their discussion of the disease. Altogether, 5 participants in the Buenos Aires group had immediate and/or extended family with ATTR-PN and 3 participants had children who had been diagnosed with ATTR-PN. Since the patient organizations recruited the participants, no identifying data were collected in order to protect privacy, and therefore demographics such as participant ages are not known. An application for Institutional Review Board approval was made to the Western Institutional Review Board, who issued an exemption. The group discussions were facilitated by a licensed psychologist experienced in conducting focus groups. We developed discussion guides for semi-structured focus groups whereby several topics were introduced but not posed as specific leading questions. Among the topics discussed were the following:

1. The patient’s experience of seeking and establishing a correct diagnosis
2. Physical or psychological symptoms experienced
3. Impact on the QOL of the patient and family

The format allowed time for participants to communicate freely about living with ATTR, and to express concerns about daily functioning, family relationships, and overall health. Participants in each group also were asked to list the symptoms of ATTR that affected their physical health and quality of life and to choose the top three that had the greatest effects on their lives.

The group discussions were transcribed. The Buenos Aires focus group was conducted in Spanish, and simultaneous translation was provided. Two members of the research team conducted a content analysis of the transcripts, using first cycle and second cycle coding techniques described elsewhere [25]. Each of the two team members independently conducted preliminary a priori coding on both transcripts based on the interview guides while noting potential patterns and themes that would likely emerge from second cycle coding. The researchers met to compare codes and discuss emerging themes. After finalizing the coding scheme, a second cycle of coding was completed and organized into larger themes representing the greatest density of responses from participants as discussed below.

## Results

Participants from both ATTR focus groups provided detailed information about what was often a long and difficult medical journey and diagnostic process. They identified a wide range of symptoms stemming from the effects of ATTR and described the major impacts the disease had on their quality of life. They talked openly about the stresses on their marital relationships and family as well as the ways in which these relationships helped them cope with the illness. Upon categorizing and codifying the disparate discussion topics, three themes most closely related to symptoms and QOL emerged: (1) diagnostic odyssey; (2) symptoms and impact; (3) family reaction and dynamics. These themes are now discussed with support from direct quotations from the focus groups negating any identifying information to protect patient confidentiality. Text in italics represent the recorded and transcribed words of the participants, included as examples of the themes identified. Brackets are used minimally for added clarification by the authors.

### Diagnostic odyssey

As with many people living with rare diseases, several participants in each of the focus groups reported enduring long periods of time searching for answers, receiving misdiagnoses, and often inappropriate or ineffective treatment before their illness was accurately diagnosed. Many primary care physicians, family care physicians, and even specialists such as community neurologists and cardiologists remain unfamiliar with ATTR and are not attuned to this disease as part of the differential diagnosis of patients’ presenting symptoms. Given that the majority of advancements in the field of ATTR diagnosis, management and care have emerged in the past decade, combined with the relative rarity and nonspecific clinical presentations of the attributable manifestations, patients continue to remain undiagnosed or misdiagnosed despite frequent medical visits and consultations with specialists. This is unfortunately not infrequent for individuals with rare diseases that present with common symptomatology, and is often described as the “diagnostic odyssey” [14, 27].

#### ATTR-CM

The diagnostic process for ATTR-CM is often long and difficult. Patients reported that they were misdiagnosed and given inappropriate treatments, sometimes multiple times.

It took them [the doctors] eight months before they came up with something out of left field. They're still off.

[I kept] going to my GP and it was "we’ll, give you a shot of testosterone." It's kind of like there's no answer. They keep trying to find out what is wrong with you. You're constantly trying to find what's wrong with you.

One patient reported retrospectively finding missed radiological signs indicative or suggestive of amyloidosis in prior test results conducted as part of an earlier workup for other unspecified reasons.

I have good medical exams that tell me it almost certainly started in 2006 because I just had some x-rays before for other reasons, and so I know pretty much when this started.

Even when there was a suspicion of ATTR from the outset, it could take time to reach diagnosis:

I made an appointment with my primary care physician and he knew my history and he gave me an EKG [electrocardiogram] and he says, "I see a blip here and I don't understand it." So, he referred me to a cardiologist. And from there, the cardiologist suspected that I had something similar to amyloidosis, so he sought his colleagues at the [research hospital] and they came back and say, "It sounds like amyloidosis, but we don't know which one it is." So, I got a referral to the [research hospital]. And I saw a hematologist; he got right to the point. He says, "Okay, any familial involvement?" I said, "My mother." He said, "I think you have hereditary." So, at that point I had all the tests and sure enough it was, that was late.

For two patients, serendipity shortened the diagnostic odyssey. They were fortunate to have a non-physician identify ATTR when their healthcare professionals had missed the diagnosis:

Tom [a friend] got word about me not being able to find out what's wrong with me…He said, "you got what I got, amyloidosis. See [a doctor who with specialized knowledge of amyloidosis]," and she [that doctor] saved my life. Whoa, she saw my chart. She said, "Who's been diagnosing you?" She said, "No, no. You're going to need a heart right off the bat. You need a heart." She went straight to the point. I was shocked, but I was happy.

I went into Afib [atrial fibrillation] and wound up going to the emergency room…they had me have an echocardiogram, and the technician who did the echocardiogram was really the one that diagnosed me, not the doc. She told the doc "this looks like, you know, the echocardiogram has that speckled appearance that is typical.” I just said, "Well, let's wait and see. It didn't go away." And finally, I heard back from the ER doc. He said, "It looks like amyloidosis."

Receiving a diagnosis of ATTR-CM did not guarantee that the patient would receive appropriate treatment:

They prescribed Metoprolol and Lisinopril, and those are not appropriate drugs for amyloidosis, and I went to various docs kind of that I knew and worked around. Finally, I realize I'm not getting anywhere with this.

Patients were actively involved in the search for answers, often using the internet as a tool. As a result, they sometimes knew more than their physicians about amyloidosis, and found a specialist themselves. One spouse was proud of her partner’s burgeoning expertise:

[He] became an amyloidosis expert by looking everything up online, and he was telling the cardiologist. His cardiologist admitted he didn't know anything about it, either.

#### ATTR-PN

Diagnosis did not come readily for patients with ATTR-PN either. Some were diagnosed rapidly because of their family history, but others did not know their family history or did not understand it. Several ATTR-PN patients were repeatedly misdiagnosed.

It took 3 years for a proper diagnosis. Doctors make incorrect diagnosis without hesitating. In [Name of city] a doctor said something was odd because I had an ulcer in my foot, and I wasn’t diabetic. I lost 3 years of treatment. That’s why I want the genetic test for my son, for him not to lose time.

I had pain in my legs and back. I thought it was my job. I saw a traumatologist and back specialist, who said I was fine, but I could not walk with the pain.

One patient had a family member who was diagnosed with ATTR, but doctors did not tell the family that the illness was heritable:

One of my cousins had symptoms and went to [name of hospital]. They said he had amyloidosis. But they didn’t say it was inherited and that all the family could have it.

Other patients knew about their family history but endured years of anxiety because they had to wait until adulthood to be tested.

I asked to have the genetic test, but I was told I should wait until I was 18. At that time, I had to wait for years, I felt very anxious about it. I got my genetic test at [hospital] and it was positive. My sister-in-law was negative, and they called to tell her. I didn’t get a call in two months, so I suspected I was positive, because we were tested at the same time. Then they came to the house with a group of psychologists, and I knew.

I wanted to test but had to wait until age 18. I got the results at, when my son was 6 months old. I would have avoided getting pregnant if I had known because my son could inherit the disease. I had to reach out to doctors, the doctors didn’t follow up on my mother or me.

Family members and patients felt that the diagnostic process was complicated by the fact that ATTR-PN manifests at different ages and under different circumstances for different family members:

My sister and I have the disease, but she had it actively, and I didn’t. They tested the tendon on our left legs and the result was not positive. Then they tested our stomachs and my sister got a positive result. I had an ulcer on my right foot. First it was just a callus, then it became a big hole. There was no solution, and it wasn’t diabetes. I got a lot of tests. Then the doctor repeated the tests and said the disease [ATTR-PN] was triggered because of my emotional situation.

Thus, patients in both ATTR groups often reportedly experienced a long and difficult diagnostic process alongside their family members. Misdiagnosis was common in both groups and as a result, deleterious delays and seemingly unnecessary or inappropriate treatment reportedly occurred. It took time and effort in both groups to find physicians with expertise in treating the illness.

### Symptoms and impact

**Fig. 1:**
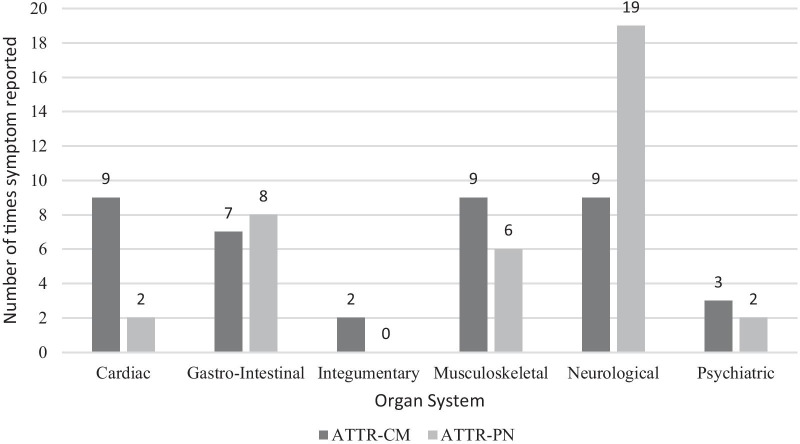
ATTR-CM and ATTR-PN reported symptoms by organ system

Because ATTR can affect multiple organ systems, it can display a variety of clinical and symptomatic characteristics [15]. ATTR-PN typically involves the sensory, motor, and autonomic nervous systems to varying degrees, so patients with ATTR-PN tend to have a wide range or mixture of symptoms [15]. Those with ATTR-CM, on the other hand, suffer from chronic and progressive heart failure as the typical pathologic manifestation. Participants were asked to identify the symptoms which had the greatest effect on their physical health, and those which had the greatest effect on their quality of life, defined as the ability to participate in the tasks and activities that were important to them. Patients with ATTR-CM reported 26 different symptoms and patients with ATTR-PN reported 24 different symptoms. Thirteen of those symptoms were reported in both groups. Table 1 lists the symptoms identified by patients and family members by organ system, and Fig. 1 shows the frequency of symptoms within each organ system for each group.

**Table 1:** Symptoms reported by organ system

Symptoms reported by organ system	ATTR-CM	ATTR-PN
*Cardiac*		
Arrhythmia	✓	✓
Atrial Fibrillation	✓	
Enlarged heart	✓	
Fluid retention/Swelling	✓	
“Hearing” own heartbeat	✓	
Increased fatigue with altitude	✓	
Intolerance to activity	✓	
Orthostatic hypotension		✓
Passing out/Fainting	✓	
Shortness of breath	✓	
*Gastro-intestinal*		
Abdominal pain	✓	
Bloating		✓
Bowel problems		✓
Changes in taste of food/loss of taste in food	✓	
Constipation	✓	✓
Diarrhea (chronic)		✓
Feeling full quickly	✓	
Loss of appetite	✓	
Low esophageal motility/ "slow digestion"		✓
Pasty feeling in mouth		✓
Upset stomach	✓	
Vomiting		✓
Weight loss	✓	✓
*Integumentary*		
Night sweats	✓	
Rash	✓	
*Musculoskeletal*		
Back pain		✓
Balance when walking	✓	
Bi lateral carpal tunnel	✓	
Clumsiness/dropping tools	✓	
Contraction of fingers	✓	
Heaviness in legs		✓
Inability to exercise	✓	
Loss of muscle mass		✓
Loss of muscle tone	✓	✓
Loss of stability		✓
Muscle twitching, cramps and spasms	✓	✓
Weakness	✓	
Weakness/knees buckling	✓	
*Neurological*		
Burning sensation in feet and hands		✓
Cold skin, hands, feet		✓
Decreased sensitivity		✓
Dizziness	✓	✓
Dry eyes		✓
Erectile dysfunction	✓	✓
Fatigue	✓	✓
General malaise	✓	✓
Hearing loss		✓
Heat intolerance	✓	
Increased sweating		✓
Lower back pain	✓	
Memory loss		✓
No sensation with full bladder		✓
Numbness in hands		✓
Pain in feet		✓
Pins and needles		✓
Reduced sexual drive	✓	
Sensitive to touch/unusual burning	✓	✓
Urinary incontinence (nocturia)		✓
Urinary retention		✓
Vision impairment	✓	✓
*Psychiatric*		
Depression	✓	✓
Mood changes	✓	✓
Sleep disorders/Insomnia	✓

#### ATTR-CM

Participants in the ATTR-CM focus group reported several features directly related to the disease’s effect on the heart including shortness of breath, atrial fibrillation, and arrhythmias. Several patients with ATTR-CM suffered from carpal tunnel syndrome. One patient with ATTR-CM experienced sharp abdominal pain, and others reported pains in their back or feet, “heavy legs” and vomiting. Male patients with ATTR-CM reported decreased sexual interest and erectile dysfunction. Mood changes and depression were widely mentioned as patients and family members faced an uncertain future and a dramatically reduced life expectancy. Several patients experienced insomnia.

Patients with ATTR-CM experienced dramatic loss of strength and stamina. They reported low energy, malaise, and “heaviness” in their limbs, ‘twitching,’ clumsiness, buckling knees, and trouble maintaining their balance. The ATTR-CM group identified intolerance to activity and inability to exercise as well as insomnia and fatigue as the most troubling symptoms they experienced. Several patients with ATTR-CM had a life-long devotion to sports and exercise that they had to curtail dramatically because of fatigue and weakness. As one spouse of an ATTR-CM patient related, “We went to Yosemite a couple years ago…it took us about fifteen minutes to go about ten feet.” The illness continually interfered with everyday tasks and with activities that brought them enjoyment. Another spouse conveyed that, “He walks our Labrador retriever every day…and he would double over from abdominal pain. It was painful to watch him.” Even the effort to put up holiday decorations could be too much. “My wife loves Christmas decorations, so I was outside trying to put the lighted candy canes in the ground and every time I'd bend over and stand up, I'd get dizzy. [It’s] just like a big effort just to stick things in the ground.”

#### ATTR-PN

Patients with ATTR-PN portrayed a wider range of symptoms than those with ATTR-CM. Patients and family members reported dysesthesias described as burning sensations or cold skin. They experienced both heightened sensitivity to touch, numbness, and lack of sensitivity. One patient had increased sensitivity in his upper body. “I couldn’t use a towel after showering because it felt like sandpaper,” but his feeling in his feet was so minimal that he had sprained his ankle without even realizing it. One patient had even experienced multiple burns because of lack of sensitivity.

Due to autonomic dysfunction, ATTR-PN patients reported constipation, diarrhea, “lazy bladders” that did not void completely, or urinary incontinence sometimes leading to multiple urinary tract infections. Some reported blockages in their digestive system, and frequent vomiting. Decreased visual or hearing acuity was also a problem for some ATTR-PN patients. Some male patients experienced decreased sexual interest and erectile dysfunction.

Several patients reportedly experienced symptoms consistent with orthostatic hypotension and others experienced dizziness. One ATTR-PN participant reported fainting. For several ATTR-PN patients, food was no longer appetizing, they lost their appetite, experienced early satiety, or frequently had an upset stomach. At one point, one participant shared that they had lost almost half their body weight, decreasing from 200 to 135 lb. Mood changes, depression and insomnia were also common in the ATTR-PN group.

ATTR-PN patients were forced to make dramatic changes in their employment and lifestyle. Two men who worked with their hands were forced to retire early because of the illness; one patient’s numbness in his hand and bent fingers made him continually drop his tools. One patient had diarrhea so severe that he had to leave his job because he did not have a bathroom nearby.

Fatigue was identified as one of the most challenging symptoms in both groups. Otherwise, the ATTR-PN group characterized gastrointestinal symptoms and sensory symptoms as having the greatest effect, which were notably different from the most impactful symptoms for the ATTR-CM group, being intolerance to activity, inability to exercise, and insomnia. It should be noted that four of the symptoms ascertained as most impactful for ATTR-PN are related to gastro-intestinal dysfunction (chronic diarrhea, weight loss, vomiting, and constipation), and an additional symptom (loss of muscle mass) is likely to be at least partially explained by digestive difficulties.

### The family system

The importance of the family system arose as a theme in several ways across the two focus groups. The illness was highly stressful for both patients and their families, and group members were open about the emotional sequelae of the illness. Spouses experienced considerable stress associated with the illness but also played a major role in coping with it. When patients had heritable forms of ATTR, they experienced stress not only from the physical effects of the illness, but also from watching their parents, children and other family members cope with the illness as well.

#### ATTR-CM

In the ATTR-CM group, the partners’ active participation in the focus groups demonstrated the critical role that caregivers play in supporting their spouse’s well-being. Spouses often took responsibility for the monitoring and management of medication. Patients and their spouses were sometimes overcome emotionally as they tried to come to terms with the effect of the disease on their lives:

You spend a lot of time in that depression/mood/mortality thing wondering what your future is going to be like.

The participants talked about the fear and anxiety spouses felt.

Right of out of the blue somebody said to us "you're going to have to have a heart transplant," and that, in 2013, maybe even still, that's a huge thing. It involves all kinds of preparation. Those of you who have had it probably understand the feelings that when you first hear about it. It's terrifying, and I was just totally knocked off balance. Crying, not knowing what are we going to do. He's too young to die. I just, it's just, so my anxiety and fear is very strong.

Speaking on my wife's behalf, she went through the same thing. When the cardiologist said to me "well, I think you're going to need a heart transplant” and she said to me in passing "can't you just wait?" I mean, it's one of those things is fear. And I said, "Hon, it's not going to get any better," but from her mind's eye, you know, maybe if you just wait longer, maybe you won't need a heart transplant, but it doesn't sound realistic, those were thoughts that caregivers go through.”

I can't sleep at night with worry and he's sleeping like a baby. Yes, the spouse, significant other, experiences extreme worry. Are you kidding me?

One wife’s anxiety was mixed with frustration over limitations in her ability to help her husband.

I worry about him, that he has all these medications he takes. He's very concerned that he does them properly, and I—I don't know how I can help make that happen. He's very organized, so I really don't worry that much about it, but I worry that his lifestyle has changed for him so much, he gets frustrated at it—and I hate to see that. I have anxiety. I want him to be well. I want to reach in and take that amyloidosis outside of his heart.

Sometimes she felt guilty: "And sometimes I feel bad that I, I'm healthy. I like being healthy.”

But patients and their caregivers adapted to the limitations and that helped them cope:

As a caregiver we tend to modify things, you know. We make it so that it works for what you're going to do. You're going to go on a hike. Well, maybe you're not going to hike ten miles. You're only going to hike one. So, you modify everything. You do that with food, as well. You don't make a big deal. You make, you know, half. So, you're only walking the one mile. You modify it so that it's not a big bone of contention.

I think we modify so that we don't have that…I can't do the Christmas lights all at one time. Maybe I'll take three days to do it. Which is fine. You try to do as much as you can and not let this… yes, it's going to change the quality of your life, but it's not going to end having quality to your life.

Family members were invariably a source of coping, motivation, inspiration and support.

[Name of patient] is a grandfather to five beautiful children and they want Pops around for many more Christmases. So, we're in it to win it.

#### ATTR-PN

In the ATTR-PN group, much of the discussion focused on the history of the illness in the family and its effects across family members. Heritable ATTR-PN is “a family disease” as one participant who had lost her husband to amyloidosis observed. Patients had witnessed parents and other relatives failing to receive an accurate diagnosis and subsequently passing away. Patients talked about their parents’, relatives’, and children’s illness in conjunction with their own. Some asymptomatic Buenos Aires participants who attended as supportive family members felt considerable anxiety over the possibility that they might test positive, or, having already tested positive, the likelihood that they might develop the disease. The greatest concern of parents who had been diagnosed with ATTR-PN was passing the illness on to their children. Some parents with ATTR-PN expressed regret over having children:

For me, it’s not hard to have a positive result, what really concerns me is my children. If I had known I had the disease, I wouldn’t have had any children.

Family members without ATTR-PN actively participated in the focus groups because they felt the impact of relatives passing away or because they were concerned about a family member who had elected not to attend the group. One group member explained her participation in this way:

My father and brother have the disease. I had a negative test result. Another brother is also negative. But today I am here with my mother because of my father and brother who are positive.

Families with ATTR-PN coped with the inevitable progression of the disease in different ways. While some families worked to ensure that other members were tested, other families preferred to postpone acquiring that knowledge.

I have a 9-year-old daughter. I don’t want her to be genetically tested, because I can’t do anything about it anyway.

In my family it was a taboo topic, although we knew we could carry the disease in the family. Two sisters died because of it. We are sure one had amyloidosis and had the genetic test done too late. Amyloidosis has marked me forever.

They were troubled by the deaths of family members they had lost to the illness and their family’s history of misdiagnosis and inadequate care.

My mother died at 61, without knowing why. All the family died young but didn’t know why.

My mother died of [the] disease when I was 17. I started with symptoms at 25. My mother was wrongly diagnosed with psychiatric problems. Now we know it was amyloidosis.

My mother died at 55. Doctors said she was crazy. She had surgeries and remained the same. They did not discover the disease.

I inherited the condition from my mother’s side. Uncles and grandparents died because of it, but without knowing why. We had wrong diagnosis. My mother was diagnosed with multiple sclerosis, and uncles with other conditions.

Yet family was also the motivation to continue to battle their illness…

My daughter is positive. I wanted to know, and I want to continue until there is a solution for her.

I'm married, two kids, 10 and 5; that's the kind of reason to stick around.

## Discussion

Although there are over 6000 rare diseases, there is, unfortunately, a common experience in the healthcare journey, which is often fraught with barriers, uncertainty, and frustration [28–30]. While a comparative analysis in the context of other rare diseases is beyond the scope of the study, this aspect is indeed both present and prevalent in ATTR, where the demands of living with and managing its effects are considerable and strenuous for patients and their families. As the unequivocal authority on the personal burden and living impact of a given condition, patients and caregivers offer critical insights and perspectives unobtainable through standard clinical means, evidenced by their growing inclusion into disease guidelines, outcomes, and recommendations [31–33]. Therefore, in order for healthcare professionals to understand how best to treat patients, and for researchers to design clinical trials that are optimally meaningful and clinically successful, we strongly believe that it is critical to better understand the patients’ and family members’ experiences and to also be aware of what aspects of the disease are most burdensome and important to those afflicted and indirectly impacted. Inclusion of the viewpoint and experience of patients and patient organizations is crucial for understanding the disease, diagnostic process, and treatment. Although employing a focus group format is not novel in rare disease, to the best of our knowledge this is the first study utilizing this method to report on the continuous and protean experiences of ATTR patients and their families [34–36].

Consistent with symptomatic information derived from previous studies, the ATTR-CM and ATTR-PN focus group patients reported experiencing a wide range of debilitating symptoms that have profoundly adverse effects on their ability to function and participate in activities of daily life [8, 14–17]. ATTR patients may develop physical complications such as pain, reduced fine motor skills and mobility, as well as mental health effects including anxiety, stress, depression, and negative feelings such as fear, hopelessness, and insecurity [24, 37, 38]. Caregivers of ATTR patients may also exhibit a high proximal burden of this disease borne from providing up to 100 h or more of patient care per week [38, 39]. In our study cohort, patients with ATTR typically experienced a long, confusing, and uncertain diagnostic odyssey in which they reported a wide range of chronic and increasingly debilitating symptoms with no explanation and often inadequate treatment. Patients with ATTR-PN often witnessed their families undergoing an arduous diagnostic odyssey, and then experiencing their own emblematic challenges. Sometimes patients did not know or understand their family history or were not told by doctors about its importance. At other times, family members realized that they themselves could have ATTR, but had to wait to be tested, or they had tested positive but had to wait to see if the disease developed. Enigmatically, accurate diagnosis was not consistently associated with appropriate and satisfactory treatment. Many patients went through multiple steps in order to find the therapeutic interventions they needed at a specialized center requiring long distance travel.

The courage and fortitude exhibited by the ATTR patients and their families reported here are exemplary of many of the people living with ATTR and their families today. The insights gathered from the collective experiences of these participants suggest several recommendations to optimally reduce many of the arduous challenges reported. Firstly, as physicians and other healthcare providers are an integral part of the patient journey, more targeted and effective education is warranted for providers who may unknowingly see ATTR patients and are unfamiliar with the early signs and symptoms of the disease in order to mitigate the likelihood that patients with ATTR undergo a protracted diagnostic odyssey. This group of first-line healthcare professionals, including neurologists, hand surgeons, cardiologists and internists/generalists may also not be aware of advances in the field of ATTR relating to diagnosis, treatment options and ongoing clinical trials. Consequently, CME programs which would increase knowledge of how to evaluate and work up a patient, and when to refer to subspecialists (e.g., heart failure cardiologists, gastroenterologists, and urologists) are also vital for the continued health and well-being of the families and the amyloidosis patients themselves. An increase in the number of specialized tertiary care centers for Amyloidosis should also be considered by medical providers, policy makers, and the patient community. Aside from direct patient impacts, enhanced knowledge and awareness in the medical community may lead to sorely needed additional studies on the ATTR patient experience. Such investigations could further aid health care providers in gaining a greater comprehension of this still inadequately understood disease and inform institutions and systems about methods to address patients’ needs and lessen their stressful medical journey. Next, patients and families would greatly benefit from ready and available access to mental health services, which their treating physicians should encourage them to use, as well as informal resources such as support groups and patient advocates who understand their unique challenges and who can provide supplementary assistance. In addition to engaging with patient support entities, it is prudent to propose efforts to increase the ease of access to, as well as awareness of, various patient advocacy groups that serve to hold professionals accountable for intervening in ways most attuned to patients’ needs. This final aspect is critical not just in the discrete patient journey but the global one as well, as these organizations aim to collectively improve the lives of patients through activities such as clinical trial guidance and recruitment, earlier diagnostic testing and identification, treatment awareness, governmental policy, and registry creation.

While the recommendations proposed here are derived from listening to the ATTR patient experience, they may also be translatable across the rare disease spectrum due to the overarching and commonly shared rare disease experience. For instance, much like ATTR, idiopathic pulmonary fibrosis (IPF) is a rare disorder which shares the difficulties of longstanding and repeated misdiagnosis and delayed interventions [40]. As with ATTR, IPF causes nonspecific cardiopulmonary symptoms, and also presents with the familiar constellation of reported health concerns, unmet medical needs, and poor QOL and outcomes characteristic of ATTR [41]. Although further expansion of this topic is outside the scope of this manuscript, it is nonetheless an interesting prospect for consideration and one which will hopefully continue to be addressed in the medical and clinical literature.

Although we believe that this preliminary real-world study offers unique and important observations regarding the journey of patients impacted by ATTR, we recognize that there are a number of study limitations or consequences that need to be considered. Most notably, the focus groups consisted of a relatively small sample of ATTR patients who were already known to patient organizations and may not be representative of all those living with ATTR, particularly those who do not participate in advocacy-type organizations. This is potentially further compounded by not knowing the participant ages, resulting in possible enrichment of a distinct subgroup of patients whose experiences are atypical of the ATTR population as a whole. As such, this patient account may not be fully representative of a larger and perhaps more heterogenous sample of ATTR patients and their families and may actually represent a more well-informed and disease-educated cohort. Additionally, outside of self-reporting, we did not attempt to reaffirm or validate health claims and reported experiences beyond what was shared with the study team via health records or direct interviews with alluded healthcare professionals and healthcare systems as this would be well beyond the scope of the intended study. We are also cognizant that some patients expressed themselves more in the focus groups than other patients, signifying that not every individual’s experience was necessarily represented equally and fully.

While future focus group studies could ultimately yield more controlled and standardized findings, these broader efforts may be impractical given limited patient access due to the rarity of the disease and the idiosyncratic quality of the conversational platform. Therefore, despite these drawbacks and their consequences, we believe that the statements from these initial small-scale ATTR focus groups provide a useful preliminary look at the lived experiences of patients with ATTR and their families and offer important insights into this and potentially other amyloidal diseases.

The results of our study may serve to provide preliminary information for a more comprehensive conceptual model of the effects of ATTR on patients’ lives. As have been previously reported, such models have the potential to elucidate the effects of a disease and help to elaborate upon the causal relationships linking symptoms, functioning, health perceptions, and quality of life [42]. A conceptual model can also importantly spur and inform the development of patient-reported outcomes (PRO) measures for use in future clinical trials and promote the design of effective treatments, goals which may ultimately materialize as the end-products of these incipient efforts [27, 43]. While no such informative models currently exist that specifically relate to ATTR, one was developed through the work of Lin and colleagues for the manifestations of another member of the amyloidosis family known as light chain (or primary) amyloidosis (AL) using published studies, patient and expert interviews, and blogs [43]. Although AL amyloidosis is a distinctly different disease caused by misfolding and deposition of immunoglobulin light chains rather than transthyretin, there are some important pathologic and symptomologic overlaps such as the potential of AL amyloid fibrils to deposit in the heart and nervous system. As more primary information on the natural history of those diagnosed and living with ATTR becomes available, we believe the future development of a conceptual model for this distinct form of amyloidosis would be merited, especially given the growing general interest in amyloidosis along with diagnostic and therapeutic advances.

Based on the results of this study it is evident that the impact of ATTR amyloidosis is substantial in both subtypes. For families with ATTR-PN, there is an increase burden of addressing the effect of the illness in multiple generations along with the dynamics of an entire extended family living at heightened future risk. The challenges faced by families living with ATTR-CM, including the emotional impact on spouses and the increased caregiving role required of them are also certainly life-altering. Therefore, to mitigate some of the adverse attributes of the disease, professionals need to develop a more sophisticated understanding of the family experience of ATTR and provide more substantive and comprehensive support to family members as well as patients. Given the social and emotional toll of TTR amyloidosis, multidisciplinary teams of healthcare professionals also need to coordinate with families because of their importance in helping patients adapt to changes in their functioning and coping strategies. Finally, patients should be encouraged to engage with patient support groups and advocacy organizations who can not only provide unique resources and a sense of communal understanding, but also elevate and amplify the voices of each individual patient so that together they can elicit the changes needed to improve the ATTR patient experience.

## Conclusion

Despite the marked and notable utilization of non-invasive diagnostic testing and a gamut of available and queued potentially disease-modifying modalities for ATTR, the overall disease experience remains laden with challenges and hurdles for both patients and caregivers. Even as the clinical understanding of ATTR continues to rapidly expand, our knowledge and awareness of the effects of the illness on patients’ lives needs to keep pace, since these inextricably linked features continue to be disparate. Patient groups remain an important resource both to support patients and their families and to provide critical information and guidance to treatment providers and clinical researchers. The stories of the patients and family members living with the illness are an essential resource for understanding how best to respond to the illness and serve to help drug development sponsors remain focused on those aspects of the disease that are meaningful and important to those directly and indirectly impacted by ATTR. Utilizing focus group reporting of lived patient and family experiences may also lead to better PROs and survey instruments that capture and represent the patient journey, as well as inform and educate healthcare professionals and the patient community. The efforts suggested here may ultimately result in a timelier diagnosis for ATTR patients and increase the potential to optimally benefit from the growing number of available and emergent therapeutic options.

## Data Availability

The data that support the findings of this study are available on request from the corresponding author DR. The data are not publicly available due to information that could compromise research participant privacy/consent.

## Abbreviations

ATTR-CM: Transthyretin Amyloid Amyloidosis with Cardiomyopathy
ATTR-PN: Transthyretin Amyloidosis with Polyneuropathy
QOL: Quality of Life
GP: General Practitioner
ATTR: Transthyretin Amyloidosis
Afib: Atrial fibrillation
EKG: Electrocardiogram
AL: Light chain amyloidosis
